# Misdiagnosis of imported *falciparum* malaria from African areas due to an increased prevalence of *pfhrp2/pfhrp3* gene deletion: the Djibouti case

**DOI:** 10.1080/22221751.2020.1815590

**Published:** 2020-09-17

**Authors:** Xavier Iriart, Sandie Menard, Pamela Chauvin, Hasna S. Mohamed, Elena Charpentier, Mohamed A. Mohamed, Antoine Berry, Mohamed H. Aboubaker

**Affiliations:** a Département de Parasitologie-Mycologie, Centre Hospitalier Universitaire de Toulouse, Toulouse, France; b Centre de Physiopathologie de Toulouse Purpan (CPTP), INSERM, CNRS, Université de Toulouse III, UPS, Toulouse, France; c Laboratoire de l’Hôpital Général Peltier, Djibouti, République de Djibouti; d Laboratoire d’analyse médicale Mer Rouge, Djibouti, République de Djibouti

**Keywords:** *Plasmodium falciparum*, histidine-rich protein 2, deletion, Djibouti, rapid diagnostic test

## Abstract

Following the diagnosis of a *falciparum* malaria case imported from Djibouti and not detected by a pfHRP2-based rapid diagnostic test (RDT), we investigated the prevalence of the *pfhrp2/pfhrp3*-deleted parasites in Djibouti using 378 blood samples collected between January and May 2019, from Djiboutian patients with suspected malaria. Malaria diagnosis by quantitative PCR confirmed the presence of *Plasmodium falciparum* for 20.9% (79/378) samples while RDTs did not detect HRP2 antigen in 83.5% (66/79) of these samples. Quantitative PCRs targeting the *pfhrp2/pfhrp3* genes confirmed the absence of both genes for 86.5% of *P. falciparum* strains. The very large number (86.5%) of f*alciparum* parasites lacking the *pfhrp2/pfhrp3* genes observed in this study, now justifies the use of non-HRP2 alternative RDTs in Djibouti. In this area and in most countries where HRP2-based RDTs constitute the main arsenal for *falciparum* malaria diagnosis, it is important to implement a systematic surveillance and to inform biologists and clinicians about the risk of malaria misdiagnosis. Further investigations are needed to better understand the mechanism of selection and diffusion of the *pfhrp2/pfhrp3*-deleted parasites.

A case of imported *falciparum* malaria acquired in Djibouti was diagnosed in 2019 in the Toulouse University Hospital (France) by microscopy and a positive *P. falciparum* specific real-time PCR (qPCR) [1]. An interesting feature of the *falciparum* strain from this patient was its inability to positivize the HRP2 band of the Rapid Diagnostic Test (RDT) (Palutop+4 Optima, Biosynex) despite a significant parasitaemia (1.3%). A *pfhrp2/pfhrp3* deletion was confirmed for this strain (data not shown).

In order to broadly evaluate the prevalence of *pfhrp2/pfhrp3*-deleted parasites in Djibouti, a study was carried out between January and May 2019. The flow chart for this study is described in Figure 1. Three hundred and seventy-eight blood samples were collected from malaria-suspected patients in 3 close sites located in Djibouti city (the Mer Rouge Medical Analysis Laboratory, the National Social Security Funds Laboratory and the Peltier Hospital Laboratory) and *Plasmodium* diagnosis was assessed with HRP2-based RDT (Palutop +4 Optima, Biosynex). Twenty-two percent (83/378) of blood samples were tested pan-pLDH positive. After extraction of DNA from the 83 pan-pLDH(+) blood samples using a DNeasy Blood & Tissue Kit® (Qiagen©), qPCR, as previously used [1], confirmed the presence of *P. falciparum* alone for 90.4% (75/83) samples, both *P. falciparum* and *P*. *vivax* for 4.8% (4/83) samples and *P. vivax* only for 4.8% (4/83) samples. Among the 79 *P. falciparum* PCR(+) samples, the RDT did not detect the *P. falciparum* HRP2 antigen for 83.5% (66/79) of samples (Table 1). Before assessing the *pfhrp2/pfhrp3* gene deletion, we performed a qPCR targeting the single copy *P. falciparum*-specific *β-tubulin* gene (PF3D7_1008700) [2]. This qPCR is less sensitive than the *pfhrp2/pfhrp3* ones with a limit of detection for *β-tubulin* qPCR of 15 parasites per µl compared to 5 and 1.67 parasites per µl for *pfhrp2* and *pfhrp3* qPCRs, respectively. The *β-tubulin* qPCR made it possible to rule out 5 *β-tubulin* PCR(-) samples that would have been false negative in *pfhrp2/pfhrp3* qPCR due to a low quantity or quality of specific DNA. Two modified qPCRs were carried out in order to amplify the exon 2 of *pfhrp2* and *pfhrp3* genes [3]. The amplification of *pfhrp2* and *pfhrp3* was performed in a final reaction volume of 10 µL containing the DNA template, with 0.15 µM of each primer (*pfhrp2-F1* and *pfhrp2-R1* for *pfhrp2* gene and *pfhrp3-F1* and *pfhrp3-R1* for *pfhrp3* gene), 5 mM or 4 mM MgCl2, respectively and LightCycler® FastStart DNA Master SYBR Green I (Roche Diagnostics GmbH). The qPCR conditions were identical for both genes with a denaturation at 95°C for 15 min, followed by 45 cycles of 95°C for 15 s, 55°C for 20 s and 72°C for 60 s. The *pfhrp2/pfhrp3* qPCRs confirmed the absence of both genes for 86.5% (64/74) of the *P. falciparum* strains (Table 1). One hundred percent of the HRP2 RDT(-) samples were deleted for *pfhrp2/pfhrp3* genes.

**Figure 1.**
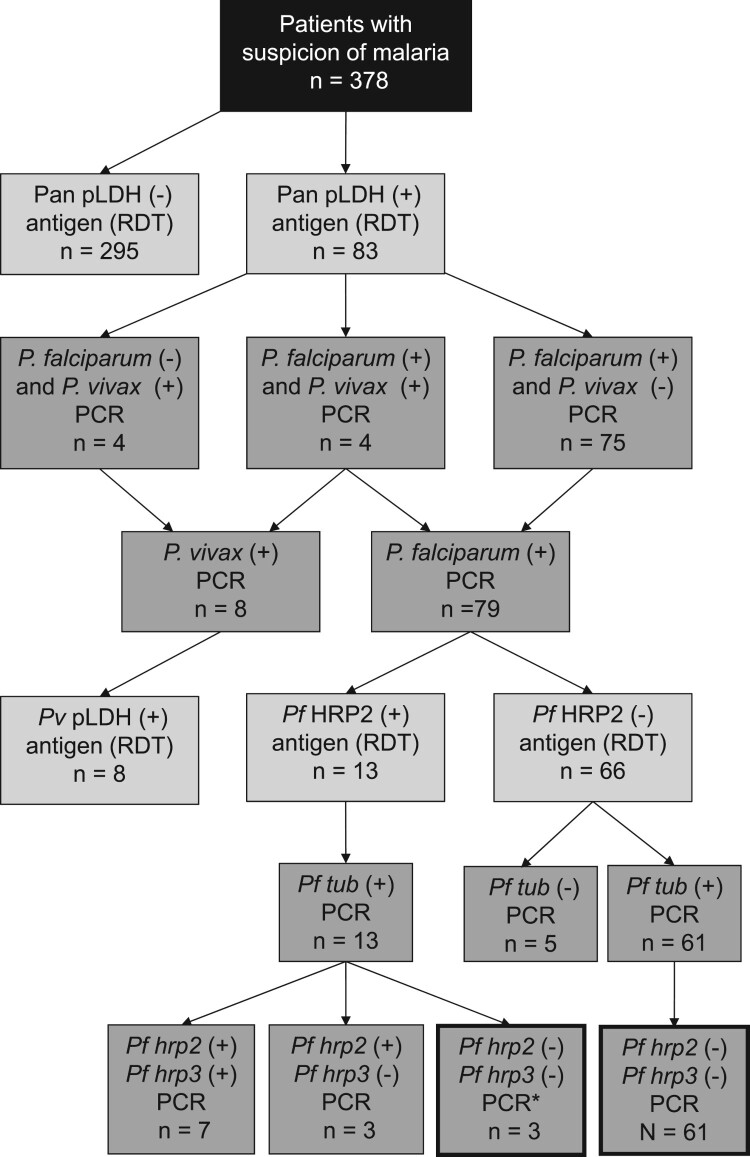
Flow chart of samples throughout the study. *Probable infection with *falciparum pfhrp2/pfhrp3* (-) strain with residual circulating HRP2 antigen from an old *falciparum pfhrp2/pfhrp3* (+) infection.

**Table 1.** Characteristics of *Plasmodium*-infected patients.

	N	%
*Plasmodium* identification by qPCR		
* Plasmodium sp.* Positive	83	
* P. falciparum* positive	79/83	95.2%
RDT results on *P. falciparum* strain		
pan-pLDH positive	79	
HRP2 negative	66/79	83.5%
*Pfhrp2/pfhrp3* genotyping on *P. falciparum* strain		
* β-tubulin* positive	74/79	93.7%
* Pfhrp2/pfhrp3* negative	64/74	86.5%

## Discussion

For more than a decade, rapid diagnostic tests (RDTs) have become essential to malaria diagnosis. A total of 276 million RDTs were sold globally in 2017, and in sub-Saharan Africa, RDTs accounted for an estimated 75% of all the diagnosis tests carried out for suspected cases [4]. RDTs can detect malaria-specific antigens: Aldolase or lactate dehydrogenase enzymes (pLDH) common to all *Plasmodium* species, LDH specific to a particular one (i.e. pvLDH specific for *P. vivax*) and histidine-rich protein 2 (pfHRP2) only produced by *P. falciparum* [5]. Due to the dominance and the severity of *falciparum* infection, 90% of the RDTs used in the diagnosis of malaria were only based on pfHRP2 detection [4]. PfHRP2-based RDTs frequently cross-react with a second *P. falciparum* protein, the PfHRP3 antigen due to a high degree of similarity in the amino acid sequence [6].

However, since 2010, false negative results with pfHRP2-based RDTs have been reported in the Amazon region and in some parts of Africa and Asia [7–9], usually due to the deletion of *pfhrp2* and/or *pfhrp3*. These *pfhrp2/pfhrp3*-deleted *falciparum* strains raise a diagnosis issue as a large number of *falciparum*-infected patients will go undetected by the pfHRP2-based RDTs. The public health consequence can be an increased risk of malaria morbidity and mortality due to delayed treatments, and an increase in the transmission of malaria.

Our study identified a very high prevalence (86.5%) of parasites lacking *pfhrp2/pfhrp3* genes among the *falciparum* strains circulating in Djibouti. The lack of previous data makes it difficult to date this trend. However, the current warning of health professionals from Djibouti, who are concerned by a high level of negative RDTs despite clinical suspicions of malaria, suggests that this trend is recent.

Djibouti is a major transit point for migrants, predominantly Afar and Somalis and lesser numbers Ethiopians and Eritreans. These human migratory and commercial flows could be at the origin of the diffusion of parasites lacking *pfhrp2/pfhrp3* genes as these strains are already present in these areas at a high frequency (4.8% and 62% in Ethiopia (2017) and Eritrea (2016), respectively) [10,11]. This data would be in favour of the importation of these clones from neighbouring countries rather than a local emergence, but this question can solely be answered through genotyping.

The second main issue is to understand how these *pfhrp2/pfhrp3*-deleted parasites are selected. The consensual assumption is the misdiagnosis and the selection of *pfhrp2/pfhrp3*-deleted strains by the exclusive use of HRP2-based tests over time, as previously observed in Eritrea [11] and predicted by mathematical modelling [12]. So, the delay or the absence of treatment against these *pfhrp2/pfhrp3*-deleted parasites due to misdiagnosis would have given them a selective advantage compared to *pfhrp2/pfhrp3* non-deleted strains. In Djibouti, malaria diagnosis is exclusively based on the use of RDT selective for the detection of *P. falciparum* and *P. vivax* but not for the *Plasmodium* species with pan-pLDH [13]. Furthermore, the low genetic diversity linked to the low transmission rate of malaria in Djibouti could have reinforced this trend [13]. Another possibility is that a *pfhrp2/pfhrp3*-deleted clone could have been indirectly selected due to the presence, on this same clone, of another mutation responsible for the resistance to an antimalarial used in this area. This scenario is unlikely in Djibouti since none of the 7 *pfhrp2/pfhrp3*-deleted strains tested in this study carried a mutation on the *PfKelch13* protein (data not shown) [14].

A systematic country-wide surveillance might ensure that the prevalence found in this study reflects the national one. However, the high prevalence of *pfhrp2*/*pfhrp3*-deleted parasites observed in Djibouti now justifies the use of non-HRP2 alternative RDTs like LDH- or aldolase-based RDTs. Indeed, the WHO recommends a policy switch to more effective WHO-approved non-HRP2 malaria tests when the prevalence of *pfhrp2*-deleted parasites meets or exceeds a 5% prevalence. Despite the costs and resources involved [15], these investments are required to control malaria morbidity and mortality in Djibouti.

In most countries, HRP2-based RDTs constitute the main diagnosis arsenal for *falciparum* imported malaria [16,17]. Consequently, a negative HRP2-based RDT should therefore not rule out a *falciparum* infection. The high frequency of mutant parasites in Djibouti raises the question on the risk of malaria misdiagnosis for locals but also for travellers or soldiers as Djibouti’s strategic situation has made it a desirable location for foreign military bases.
